# Universality and diversity in the signal transduction pathway that regulates seasonal reproduction in vertebrates

**DOI:** 10.3389/fnins.2014.00115

**Published:** 2014-05-21

**Authors:** Yusuke Nakane, Takashi Yoshimura

**Affiliations:** ^1^Laboratory of Animal Physiology, Department of Applied Molecular Biosciences, Graduate School of Bioagricultural Sciences, Nagoya UniversityNagoya, Japan; ^2^Institute of Transformative Bio-Molecules (WPI-ITbM), Nagoya UniversityNagoya, Japan; ^3^Avian Bioscience Research Center, Graduate School of Bioagricultural Sciences, Nagoya UniversityNagoya, Japan; ^4^Division of Seasonal Biology, Department of Environmental Biology, National Institute for Basic BiologyOkazaki, Japan

**Keywords:** circadian rhythm, pars tuberalis, saccus vasculosus, deep brain photoreceptor, thyroid-stimulating hormone, thyroid hormone, cerebrospinal fluid-contacting neuron, coronet cell

## Abstract

Most vertebrates living outside the tropical zone show robust physiological responses in response to seasonal changes in photoperiod, such as seasonal reproduction, molt, and migration. The highly sophisticated photoperiodic mechanism in Japanese quail has been used to uncover the mechanism of seasonal reproduction. Molecular analysis of quail mediobasal hypothalamus (MBH) revealed that local thyroid hormone activation within the MBH plays a critical role in the photoperiodic response of gonads. This activation is accomplished by two gene switches: thyroid hormone-activating (DIO2) and thyroid hormone-inactivating enzymes (DIO3). Functional genomics studies have shown that long-day induced thyroid-stimulating hormone (TSH) in the pars tuberalis (PT) of the pituitary gland regulates DIO2/3 switching. In birds, light information received directly by deep brain photoreceptors regulates PT TSH. Recent studies demonstrated that Opsin 5-positive cerebrospinal fluid (CSF)-contacting neurons are deep brain photoreceptors that regulate avian seasonal reproduction. Although the involvement of TSH and DIO2/3 in seasonal reproduction has been confirmed in various mammals, the light input pathway that regulates PT TSH in mammals differs from that of birds. In mammals, the eye is the only photoreceptor organ and light information received by the eye is transmitted to the pineal gland through the circadian pacemaker, the suprachiasmatic nucleus. Nocturnal melatonin secretion from the pineal gland indicates the length of night and regulates the PT TSH. In fish, the regulatory machinery for seasonal reproduction, from light input to neuroendocrine output, has been recently demonstrated in the coronet cells of the saccus vasculosus (SV). The SV is unique to fish and coronet cells are CSF-contacting neurons. Here, we discuss the universality and diversity of signal transduction pathways that regulate vertebrate seasonal reproduction.

## Introduction

Animals that reproduce year-round (e.g., human beings and mice) are so-called non-seasonal breeders. However, in most animals living outside of tropical zones, gametogenesis occurs during a particular period of the year. This allows the animals to produce offspring in a favorable season. Such animals are called seasonal breeders. The timing of the breeding period is related to the length of the gestation or incubation period. Animals that mate in spring-summer (e.g., hamsters, quail, and medaka) are called long-day breeders, whereas those that mate in fall-winter (e.g., sheep, emu, and salmon) are called short-day breeders.

## Involvement of the mediobasal hypothalamus in the regulation of seasonal reproduction in birds

The photoperiodic responses of seasonally breeding birds are so robust and rapid that they provide excellent models for the study of seasonal reproduction. Avian gonads change size seasonally, increasing or decreasing more than one hundred-fold within a few weeks. For example, when Japanese quail (*Coturnix japonica*) kept under short-day conditions are transferred to long-day conditions, an increase in plasma gonadotropin (luteinizing hormone: LH) concentration is observed by the end of the first long day and spermatogenesis is accomplished within 2 weeks (Nicholls et al., 1983). Because quail can be readily obtained from quail farms, it has been frequently used for the study of photoperiodism. Quail has been used as a model to explore the center that regulates seasonal reproduction. Lesions of the mediobasal hypothalamus (MBH), including the median eminence (ME) and infundibular nucleus (IN), or the dorsal MBH result in low plasma LH concentration and attenuate testicular growth under long-day conditions (Sharp and Follett, 1969; Davies and Follett, 1975). Electrical stimulation of the MBH increases plasma LH concentration (Konishi et al., 1987) and testicular growth (Ohta et al., 1984). Birds are receiving light information within the brain and local illumination of the MBH induces testicular development, suggesting the presence of deep brain photoreceptors within the MBH (Homma et al., 1979). In addition, expression of the neuronal activation marker, c*-Fos*, was observed within the ME and IN in response to a single long-day stimulus (Meddle and Follett, 1995, 1997). Therefore, the MBH is considered to be the center for seasonal reproduction in birds.

## Local thyroid hormone activation driven by pars tuberalis thyrotropin is the key for eliciting photoperiodic response in birds

**Figure 1:**
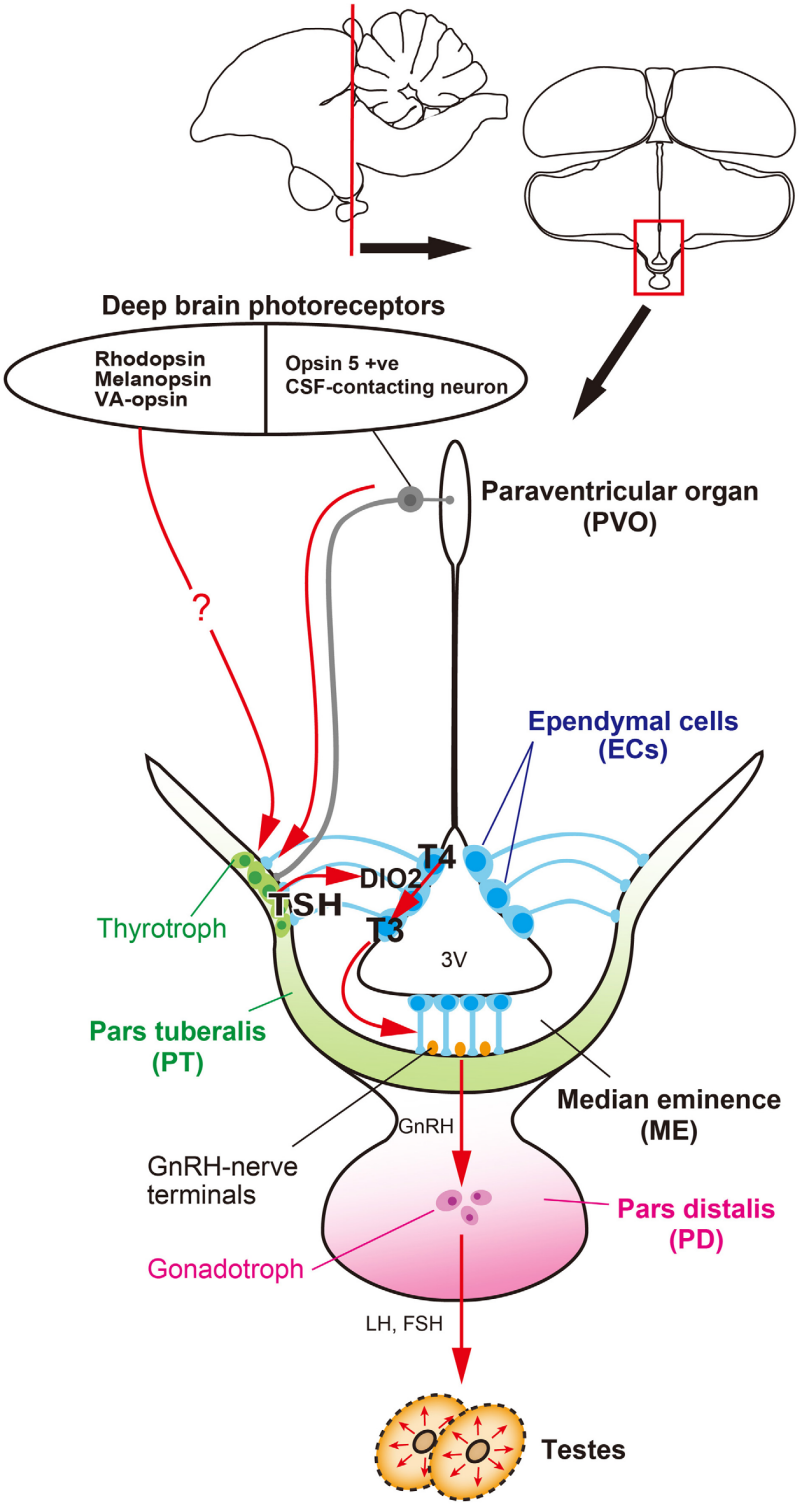
**Signal transduction pathway regulating seasonal reproduction in birds**. Light information received by deep brain photoreceptors is transmitted to the pars tuberalis (PT) of the pituitary gland, a regulatory hub for seasonal reproduction. Long day-induced thyrotropin (TSH) in the PT acts on ependymal cells to induce a thyroid hormone-activating enzyme, DIO2. The bioactive thyroid hormone, T_3_ is converted by DIO2 from the prohormone, T_4_. T_3_ regulates seasonal morphological changes in GnRH nerve terminals and glial processes, thereby regulating or modulating GnRH secretion.

Lack of genome information had been a barrier to avian research for long time. However, differential subtractive hybridization analysis has revealed that long-day stimuli induce mRNA that encode type 2 deiodinase (DIO2) in the ependymal cells (ECs) (also known as tanycytes) lining the ventro-lateral walls of the third ventricle within the MBH (Yoshimura et al., 2003) (Figure 1). DIO2 is a thyroid hormone-activating enzyme that converts prohormone thyroxine (T_4_) to bioactive 3,5,3'-triiodothyronine (T_3_). Subsequently, long day suppression of type 3 deiodinase (DIO3) was reported. DIO3 is a thyroid hormone-inactivating enzyme that converts T_4_ and T_3_ to inactive metabolites rT_3_ and T_2_. These reciprocal gene switches, *DIO2/3*, appear to be the key for regulation of seasonal reproduction in quail (Yasuo et al., 2005). Indeed, T_3_ was up-regulated by these gene switches in the MBH under long-day conditions. In addition, ICV administration of T_3_ mimicked long day-induced testicular growth under short-day conditions and infusion of DIO2 inhibitor blocked testicular growth under long-day conditions (Yoshimura et al., 2003). It is well established that thyroid hormone is essential for brain development and is also critical for adult brain plasticity (Bernal, 2005). Indeed, T_3_ is reported to cause morphological changes in gonadotropin-releasing hormone (GnRH) nerve terminals and glial cells in the ME (Yamamura et al., 2006). Most GnRH nerve terminals are covered with glial cells and do not touch the basal lamina of the perivascular space of portal capillaries under short-day conditions. Under long-day conditions, however, many GnRH nerve terminals are in direct contact with the basal lamina. T_3_ implantation under short-day conditions mimics these morphological changes and results in testicular development (Yamamura et al., 2006). These findings suggest that local activation of thyroid hormone within the MBH is a critical event for the seasonal regulation of GnRH secretion.

The availability of genome sequences in avian species has provided an opportunity to employ a functional genomics approach to photoperiodism research. Using a functional genomics approach, long-day induction of *TSHB* mRNA, which encodes the β subunit of thyroid-stimulating hormone (TSH), was observed in the par tuberalis (PT) of the pituitary gland. This *TSHB* induction preceded *DIO2/3* switching by about 4 h. Localization of TSH receptor (TSHR) was observed in the ECs where *DIO2/3* are expressed, suggesting that PT TSH may act on the TSHR expressed in the ECs to regulate *DIO2/3* switching. Indeed, ICV infusion of TSH drives *DIO2/3* switching and testicular growth, even under short-day conditions (Nakao et al., 2008) (Figure 1). However, the transport system of PT TSH to the ECs remains unclear.

## Involvement of deep brain photoreceptors in avian seasonal reproduction

Although the eye is the only photoreceptor organ in mammals, photoreceptive organs in non-mammalian vertebrates include eyes, pineal organs, and deep brain photoreceptors. Photo-capability in the deep brain was first demonstrated in European minnows, in which it controls changes in skin color (von Frisch, 1911). Subsequently, evidence of a deep brain photoreceptor that regulates seasonal reproduction in ducks was reported. Blind ducks continue to show photoperiodic responses, whereas enveloping the heads of ducks with black caps blocks testicular responses (Benoit, 1935). Moreover, injection of India ink under the scalp in pinealectomized sparrows abolishes the photoperiodic response (Menaker et al., 1970). Both pinealectomized and blinded quail are reported to undergo gonadal development in response to light cues (Siopes and Wilson, 1974). In addition, photo-stimulation of the hypothalamus using light fiber and light-emitting beads prompts testicular development in sparrows (Yokoyama et al., 1978) and Japanese quail (Homma et al., 1979). It has been confirmed that a broad spectrum of light penetrates into the brains of various vertebrate species (Hartwig and van Veen, 1979; Foster and Follett, 1985; Oishi and Ohashi, 1993).

**Figure 2:**
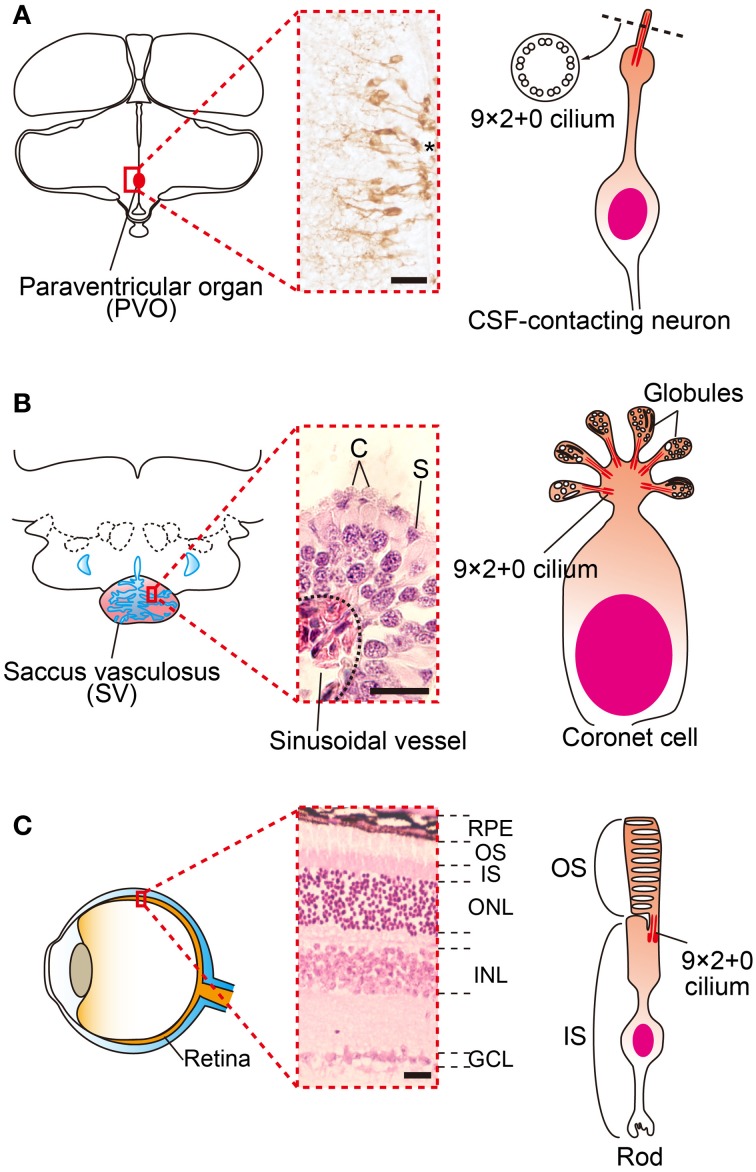
**Family of cerebrospinal fluid (CSF)-contacting neurons. (A)** Schematic drawings and picture of OPN5 positive CSF-contacting neurons in the paraventricular organ (PVO) of quail. **(B)** Schematic drawing and picture of coronet cells in the salmon saccus vasculosus (SV). The SV consists of coronet cells (C) and supporting cells (S). Globules of coronet cells are based on 9 × 2 + 0 cilia. **(C)** Schematic drawing and picture of a mammalian retina and photoreceptor. The outer segments of rod and cone cells are also based on 9 × 2 + 0 cilia. ^*^third ventrile. RPE, retinal pigment epithelium, OS, outer segment, IS, inner segment, ONL, outer nuclear layer, INL inner nuclear layer, GCL, ganglion cell layer. Scale bars indicate 20 μm.

Many groups have tried to identify deep brain photoreceptors. Several rhodopsin family proteins (e.g., rhodopsin (RH), melanopsin (OPN4), and vertebrate ancient (VA)-opsin) were reported to be localized in the avian deep brain region (Silver et al., 1988; Wada et al., 1998; Chaurasia et al., 2005; Halford et al., 2009). In addition, a novel opsin called Opsin 5 (OPN5: also known as neuropsin) was recently reported to be localized in the paraventricular organ (PVO) within the MBH (Nakane et al., 2010; Yamashita et al., 2010). This is intriguing because lesions around the PVO block the photoperiodic responses of gonads in Japanese quail (Sharp and Follett, 1969). Immunohistochemical analysis of OPN5 revealed its presence in the cerebrospinal fluid (CSF)-contacting neurons (Figure 2A). The CSF-contacting neurons in the PVO have long been a candidate deep brain photoreceptor because the retina and pineal organ evaginate from the diencephalon (around the third ventricle where the PVO is located) and the CSF-contacting neurons resemble photoreceptor cells in the developing retina (Vigh-Teichmann et al., 1980). Functional analysis demonstrated that OPN5 is a short-wavelength sensitive photopigment (Nakane et al., 2010; Yamashita et al., 2010) and long-day stimulation with short-wavelength light triggered testicular growth in eye-patched and pinealectomized quail (Nakane et al., 2010). Therefore, OPN5-expressing, CSF-contacting neurons in the PVO may be deep brain photoreceptors that are important for seasonal reproduction in birds (Figures 1, 2A).

In summary, a series of quail studies have uncovered the signal transduction cascade that regulates seasonal reproduction, from photoreceptors to neuroendocrine output, in birds (Figure 1). That is, light information received by deep brain photoreceptors (e.g., OPN5, RH, OPN4, VA-opsin, etc.) is transmitted to the PT and long-day induced TSH secreted from the PT acts on TSHR to regulate DIO2/3 switching in the ECs. Bioactive T_3_ converted from T_4_ by DIO2 causes morphological changes in GnRH nerve terminals and glial processes in the ME, thereby regulating seasonal changes in GnRH secretion.

## Signal transduction cascade for seasonal reproduction in mammals

**Figure 3:**
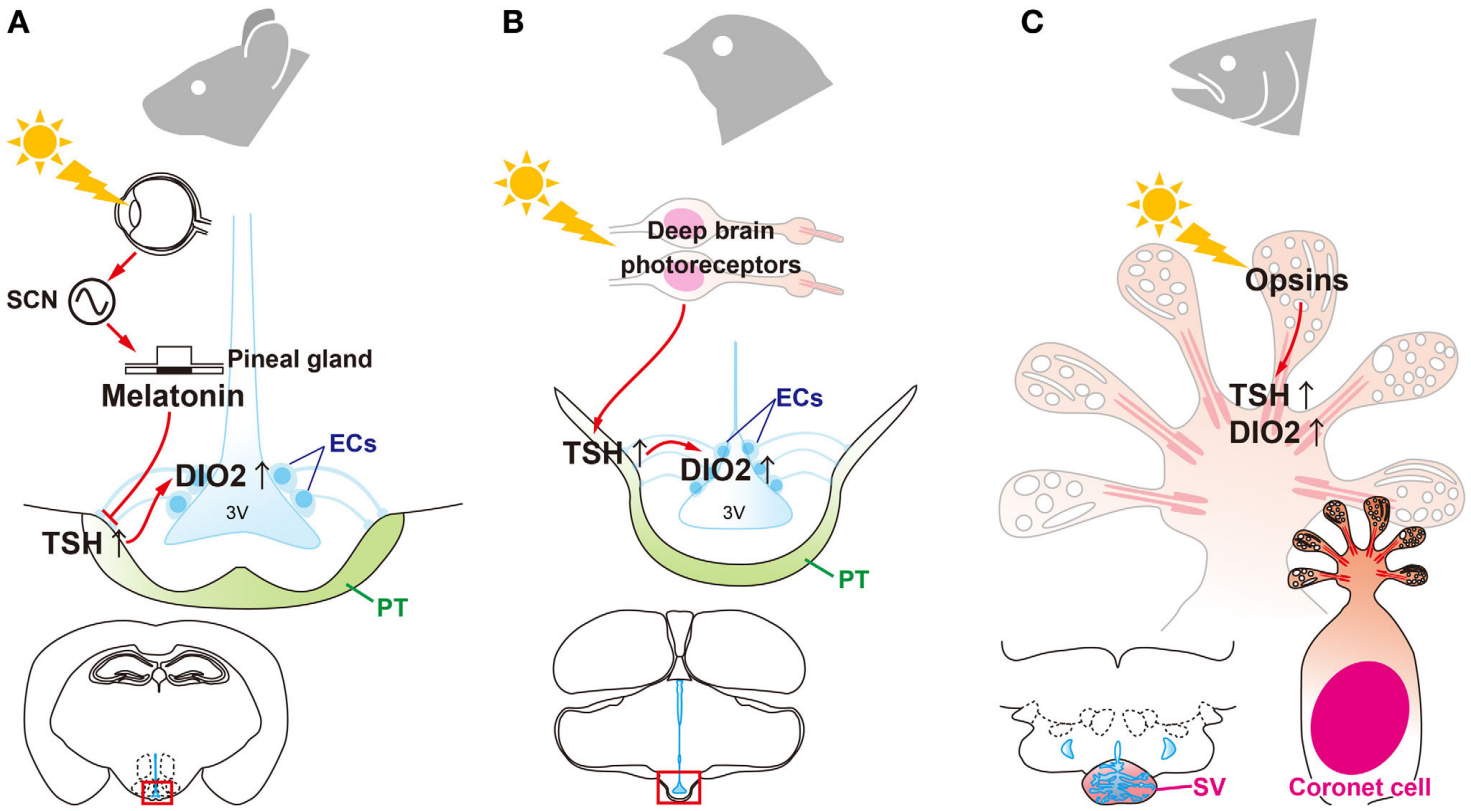
**Universality and diversity of signal transduction pathways that regulate seasonal reproduction in vertebrates. (A)** Eyes are the only photoreceptor organ in mammals. Light information is transmitted through the suprachiasmatic nucleus (SCN) to the pineal gland. Photoperiodic information is encoded by the pattern of melatonin secretion from the pineal gland. Melatonin regulates the “springtime hormone,” TSH, in the pars tuberalis (PT) of the pituitary gland. **(B)** In contrast to mammals, light information is directly received by deep brain photoreceptors in birds and is then transmitted to the PT to induce TSH. **(C)** In fish, all of the machinery required for seasonal reproduction (from photoreceptors to neuroendocrine output) is located in the saccus vasculosus (SV).

Thyroidectomy blocks the transition of seasonal reproductive state in sheep (Moenter et al., 1991), and it has been known for several decades that thyroid hormone is involved in the regulation of mammalian seasonality (Nicholls et al., 1988). However, its precise mode of action was unknown. After the discovery of photoperiodic *DIO2/3* switching in birds, photoperiodic regulation of *DIO2* and/or *DIO3* within the MBH was reported in a number of mammalian species, such as hamsters (Watanabe et al., 2004, 2007; Revel et al., 2006; Barrett et al., 2007; Freeman et al., 2007; Yasuo et al., 2007a), rats (Yasuo et al., 2007b), mice (Ono et al., 2008) and even in short-day breeding sheep (Hanon et al., 2008) and goats (Yasuo et al., 2006). Therefore, local thyroid hormone activation within the MBH is considered to be central in the regulation of seasonal reproduction in mammals (Figure 3). However, in marked contrast with birds, the eye is the only photoreceptor organ. Light information is transmitted to the pineal gland through the circadian pacemaker, the suprachiasmatic nucleus (SCN). In mammals, photoperiodic information is decoded based on the duration of melatonin secretion by the pineal gland (Reiter, 1980; Yamazaki et al., 1999). Therefore, pinealectomy abolishes seasonal responses and melatonin administration mimics the effect of short photoperiod in mammals. Thus, melatonin is considered to play a deterministic role in mammalian seasonal reproduction (Reiter, 1980). Although melatonin controls *DIO2/3* switching, melatonin receptors are absent in the ECs where *DIO2/3* are expressed (Schuster et al., 2000; Song and Bartness, 2001). In contrast, melatonin receptors are densely expressed in the PT (Williams and Morgan, 1988; Wittkowski et al., 1988; Reppert et al., 1994; Klosen et al., 2002; Dardente et al., 2003). Therefore, it was predicted that TSH secreted from the PT may mediate the melatonin action to regulation of *DIO2/3* switching in mammals. This hypothesis was tested using TSHR and melatonin receptor knockout mice (Ono et al., 2008; Yasuo et al., 2009). Melatonin administration had no effect on *DIO2/3* switching in the TSHR and MT1 melatonin receptor null mice, whereas melatonin affected *DIO2/3* switching in MT2 null mice. This suggests that melatonin acts on the MT1 melatonin receptor to regulate *DIO2/3* switching through the TSH-TSHR signaling pathway in mammals (Figure 3).

The RF-amides such as kisspeptin, a ligand for the G protein coupled receptor, GPR54, and RFamide-related peptide 3 (RFRP-3) are involved in the regulation of GnRH secretion (Clements et al., 2001; Kotani et al., 2001; Muir et al., 2001; Ohtaki et al., 2001; Clarke et al., 2008). Seasonal regulation of kisspeptin and RFRP-3 has been reported in hamsters (Revel et al., 2006, 2008). Administration of TSH to Djungarian and Syrian hamsters induces the expression of kisspeptin and RFRP-3 as well as gonadal development under short-day conditions (Klosen et al., 2013). T_3_ also provoked significant testicular growth and kisspeptin expression in Siberian hamsters (*Phodopus sungorus*) under short-day conditions (Henson et al., 2013). This suggests that long-day induces TSH and, following the activation of thyroid hormone by DIO2, regulates kisspeptin, RFRP-3 and the hypothalamic-pituitary-gonadal (HPG)-axis in mammalian species.

## Signal transduction cascade for seasonal reproduction in fish

Fish also show marked seasonal changes in physiology and behavior. Medaka (*Oryzias latipes*), are long-day seasonal breeders, and their gonads develop in response to elongated day-length (Koger et al., 1999). Salmonids, short-day seasonal breeders, show distinct photoperiodic responses, such as migration and parr-smolt transformation. Smoltification is closely linked to thyroid hormone (Robertson, 1949; Nishikawa et al., 1979). Although all fishes examined have had higher circulating levels of melatonin during the night than during the day, there are few reliable data consistent with a major physiological role for melatonin in the seasonal reproduction of fish (Urasaki, 1976; Garg, 1989; Masuda et al., 2005; Borg, 2010). This is in marked contrast to mammals, but is similar to birds. Fish do not have anatomically distinct PTs, a regulatory hub of seasonal reproduction in birds and mammals. Thus, the signal transduction pathway for fish seasonal reproduction remains unknown.

A recent study of masu salmon (*Oncorhynchus masou masou*) revealed that key elements for vertebrate seasonal reproduction, such as photopigments, TSH, TSHR, and DIO2, are expressed in the saccus vasculosus (SV). The SV is an organ only observed in fish and is located at the floor of the hypothalamus, posterior to the pituitary gland. Although its existence was first described in the 17th century (Collins, 1685), its physiological function remained a mystery for several centuries. In the SV, a folded EC layer makes a chamber that is directly connected to the third ventricle. Abundant sinusoidal vessels cover the whole external surface of the SV. The EC layer of the SV mainly consists of coronet cells and supporting cells (Sueiro et al., 2007). The coronet cells have morphologically specialized features; globules occupy the apical cellular structures of these cells (Figure 2B). Each globule has 9 × 2 + 0 cilia, as do photoreceptors in the retina (Figure 2C) and CSF-contacting neurons in the PVO (Figure 2A). The coronet cells also possess manifold primary vesicles (Jansen et al., 1982; Vigh and Vigh-Teichmann, 1998). Therefore, the coronet cells are considered to be CSF-contacting neurons.

Immunohistochemical analysis has revealed localization of photopigments (OPN4 and SWS1), TSH, and DIO2 in coronet cells (Nakane et al., 2013). The expression of these photoperiodic regulatory mechanisms within the SV implies that the SV plays a pivotal role as a seasonal sensor in fish. Indeed, isolated SVs respond to photoperiodic changes in *in vitro* and ablation of the SV prevents photoperiodically-induced gonadal development (Nakane et al., 2013). This suggests that coronet cells have multiple functions, including photoreception and neuroendocrine output (Figure 3).

## Conclusion remarks

The mechanisms of seasonal time measurement were a mystery for long time. However, recent studies have uncovered the signal transduction pathway that regulates seasonal reproduction in birds, mammals, and fish. These studies revealed the universality (i.e., signal transduction machineries) and diversity (responsible cells or organs) of these mechanisms among vertebrate species (Figure 3). This is similar to the structural and functional evolution of the pineal organ (Korf, 1994; Falcón et al., 2009). In non-mammalian vertebrates, the pinealocyte contains photoreceptors, the circadian clock, and neuroendocrine output in the form of melatonin. In marked contrast with non-mammalian vertebrates, the mammalian pinealocyte is specialized as a neuroendocrine organ for melatonin secretion. This is why the pineal organ is generally referred to as the pineal gland in mammals. As expressed by Ernst Haeckel's phrase “ontogeny recapitulates phylogeny,” the rat pineal gland responds to light during the postnatal period (Zweig et al., 1966; Tosini et al., 2000; Fukuhara and Tosini, 2003). Because multi-functionality is considered to be a general feature of ancient cell types (Arendt, 2008), coronet cells appear to be the ancestral vertebrate seasonal sensors.

### Conflict of interest statement

The authors declare that the research was conducted in the absence of any commercial or financial relationships that could be construed as a potential conflict of interest.
